# The Diamond League athletic series: does the air quality sparkle?

**DOI:** 10.1007/s00484-021-02114-z

**Published:** 2021-03-24

**Authors:** James R. Hodgson, Lee Chapman, Francis D. Pope

**Affiliations:** Present Address: School of Geography, Earth and Environmental Sciences, University of Birmingham, B15 2TT, Birmingham, UK

**Keywords:** Diamond League, Athletics, Meteorology, Air quality, Physical health, Exercise performance

## Abstract

Urban air pollution can have negative short- and long-term impacts on health, including cardiovascular, neurological, immune system and developmental damage. The irritant qualities of pollutants such as ozone (O_3_), nitrogen dioxide (NO_2_) and particulate matter (PM) can cause respiratory and cardiovascular distress, which can be heightened during physical activity and particularly so for those with respiratory conditions such as asthma. Previously, research has only examined marathon run outcomes or running under laboratory settings. This study focuses on elite 5-km athletes performing in international events at nine locations. Local meteorological and air quality data are used in conjunction with race performance metrics from the Diamond League Athletics series to determine the extent to which elite competitors are influenced during maximal sustained efforts in real-world conditions. The findings from this study suggest that local meteorological variables (temperature, wind speed and relative humidity) and air quality (ozone and particulate matter) have an impact on athletic performance. Variation between finishing times at different race locations can also be explained by the local meteorology and air quality conditions seen during races.

## Introduction and background

Poor urban air quality (UAQ) is a serious worldwide environmental issue with detrimental impacts on human health and the wider environment (European Commission 2017a, 2017b; Kampa and Castanas 2008; Lim et al. 2012; Walton et al. 2015). Along with premature deaths, shorter-term effects including cardiovascular and respiratory distress and irritation are common in the wider populations, and heightened for those with preexisting conditions (Burnett et al. 2014; Lelieveld et al. 2015). The main pollutants concerning human health are nitrogen dioxide (NO_2_), ozone (O_3_) and particulate matter (PM: Particles with a diameter of 10 μm (PM_10_) and 2.5 μm (PM_2.5_) or less, Rajagopalan et al. 2018; Sun and Zhu 2019). As well as short-term irritation of the nose, mouth, throat and cardio-respiratory systems, these pollutants, along with others, can lead to cardiovascular and respiratory diseases, reduced lung function and asthma (Burnett et al. 2014; EEA 2013; Lelieveld et al. 2015). Recent work has also highlighted negative impacts of UAQ upon cognitive level (Calderon-Garciduenas et al. 2016; Clifford et al. 2016; Shehab and Pope 2019; Sunyer et al. 2015).

In extreme cases, the negative impacts of UAQ could outweigh the positive impacts of exercise (Guo et al. 2020; Strak et al. 2010; Tainio et al. 2016. Consequently, with encouragement for greater levels of exercise and active transport to combat a global obesity crisis and pollution: there is a likelihood of a greater proportion of society being exposed to poor, albeit improving, UAQ (COMEAP 2009, 2010; Devarakonda et al. 2013; Kobayashi et al. 2017; Sallis 2008; Shugart 2016).

In contrast to recreational exercisers who are largely free to choose when they exercise, elite athletes and professional sport-people are constrained to set competition times, potentially resulting in them performing in nonideal environmental conditions. Although at some landmark events, such as the now rearranged 2021 Tokyo Olympic Games, start times of some events are scheduled to avoid the most detrimental meteorological conditions (BBC Sport 2019a, 2019b).

The International Association of Athletics Federations has held a season-long track and field athletics series known as Diamond League since 2010, with plans to continue developing the series in the future (IAAF 2019). With events taking place in multiple European locations and additional international locations, Diamond League provides a global case study of the impact of local meteorological and air quality conditions on elite athletes, something that has rarely been studies outside of laboratory conditions (Giles and Koehle 2014). In this paper we assess the impacts of meteorological variables (temperature, relative humidity, heat stress and wind speed) and air quality (O_3_, NO_2_ and particulate matter in the PM_2.5_ and PM_10_ size fractions) upon athletic performance using a statistical approach.

### Meteorological impacts on performance

Meteorological impacts on performance are often anecdotal, but a number of laboratory and marathon studies have shown that elevated temperatures over 9.9°C decrease performance (Ely et al. 2007; Helou et al. 2012; Vihma 2010; Vugts 1997). This is due to alteration in circulatory, endocrine, and thermoregulatory systems during exercise to reduce the likelihood of negative effects caused by increased internal body temperatures (Casa 1999; Miller-Rushing et al. 2012; Nadel 1990). Internal body temperature increases and can result in dehydration, hyperthermia and heat stress and occur due to the cardiovascular system giving precedence to maintaining blood flow to vital organs during exercise (Casa 1999; Nadel 1990). Consequently, the rise in internal body temperatures results in higher blood lactate levels within contracting muscles and reduced maximal oxygen uptake, contributing to fatigue and reduced power output of functioning muscles (Nybo et al. 2014; Zhao et al. 2013; Miller-Rushing et al. 2012). Reduced temperatures can limit internal core temperatures and improve performances to an extent, although extreme cold results in reduced blood supply and cardiorespiratory capacity (Oksa et al. 2004; Weller et al. 1997). Marathon performances have often been examined in relation to the impact of temperature on competitors, with several studies confirming that increased temperatures result in slower finishing times and determining that temperatures in the range of 3.8–9.9°C are ideal (Helou et al. 2012; Vugts 1997).

After temperature, relative humidity has been identified to be the next most influential meteorological variable on performance (Bigazzi 2017). Reduced heat dissipation under high humidity levels results in difficulty in maintaining optimum core body temperatures as previously examined, with the negative impacts of which being identical (Casa 1999; Helou et al. 2012; Nadel 1990). The combined influence of increased temperatures and relative humidity, otherwise termed heat stress, can also heighten the risk to health, as well as athletic performance, due to the combined stress this puts on the body (Maughan et al. 2007a).

Wind direction, speed and chill can influence performance. Head- and tail-winds are likely to reduce and improve performance, respectively, due to increased resistance or additional propulsion (Davies 1980). However, this has not always been shown in previous research due to variability in wind directions experienced during the race and the lapped nature of many events, particularly those held on athletics tracks (Vihma 2010). In events which have both head- and tail-winds, it is likely that the former will be more detrimental than the latter is beneficial (Davies 1980). Similarly, the cooling effect of wind can help maintain optimal core temperatures in elevated temperatures but can lead to reduced performance in colder conditions as blood flow is diverted from contracting muscles to help maintain core temperatures and vital functions (Maughan et al. 2007a, b). Finally, Hodgson et al. (preprint), determined that local meteorology, particularly a combined influence of increased temperature, relative humidity, and wind speeds, could be detrimental to the performance of the general public’s performance in timed 5 km events (parkrun).

### Air quality impacts on performance

The majority of research on air quality and athletic performance has been conducted in highly controlled laboratory settings to allow for greater control of variables (synthesised in Giles and Koehle 2014). Although findings are mixed, there is agreement that higher intensity exercise sees an increased potential for pollution uptake due to a switch from nasal to oral breathing and reduced respiratory defences (Giles and Koehle 2014; Muns et al. 1995; Niinimaa et al. 1980; Ultman et al. 2004). Also, research has shown that increases from an average walking speed of 2–6 km/h to jogging and running and cycling 10 km/h quicker can more than double the inhalation dose of pollutants due to increased inhalation and exposure to pollution (Bigazzi 2017; Lichter et al. 2017).

Several studies have highlighted that O_3_ can reduce performance, likely due to reduced lung function and irritant qualities (Carlisle and Sharp 2001; McKenzie and Boulet 2008; Rundell and Caviston 2008; Rundell 2012). This irritant quality of O_3_ and other pollutants can trigger asthma attacks, a common respiratory condition in both the general public and elite athletes, with exercise also enhancing the negative impacts of pollution (Folinsbee et al. 1994; Lippi et al. 2008; McCreanor et al. 2007; McKenzie and Boulet 2008; Rundell 2012; Weinmann et al. 1995). The same has been shown with PM exposure impacting on lung function (Cutrufello et al. 2011; Rundell and Caviston 2008; Rundell et al. 2008; Rundell 2012). Furthermore, research has shown that preexposure to pollution, as well as exercise performance in polluted conditions, can reduce VO_2_ max (Florida-James et al. 2011; Kargarfard et al. 2015).

As with real-world examination of meteorological effects, little work on the actual impact of air quality on athletic performance has been conducted. Of this, the most notable is that of Marr and Ely (2010) and Helou et al. (2012) who both examined yearly marathon finishing times. Results suggest that PM_10_ is detrimental to female performance whilst O_3_ is the most common inhibitor to quick finishing times. This latter result corresponds well with the findings of Hodgson et al. (preprint) and the examination of parkrun finishing times over a six year period. However, both studies noted that the reduction in performance under elevated O_3_ levels is likely tied to the commonly associated temperature increases, as well as the pollutants’ irritant qualities. Additionally, Hodgson et al. (preprint) showed in a number of instances that higher NO_2_ concentrations saw improved performances, again likely tied to the O_3_-VOC-light-NO_x_ chemical reaction and reduced temperatures. The influence of PM was often unclear or insignificant, although this is thought to be due to the highly spatially variable nature of the pollutant and distances between monitoring sites and parkrun events. To support this, a long-term study of the German professional football league has shown a causal relationship between local PM levels and player productivity (Lichter et al. 2017).

In summary, there has been limited real-world examination of the impacts of meteorology and/or air quality on athletic performance, and what has been performed has largely been focused on elite marathon competitors (Helou et al. 2012; Marr and Ely 2010; Vihma 2010). However, our recent study examined the impact of both on the general public over 5-km events, highlighted the need to recognise that meteorology and air quality and not separate parameters for investigation (Hodgson et al. preprint). For example, the aforementioned variations in O_3_ and NO_2_ levels in response to elevated temperatures and sunlight and the impact of relative humidity and wind speed on PM. Consequently, meteorology, and by further extension, climate, and air quality are intrinsically linked, with the combined effect of elevated temperatures and pollution contributing to an approximate 423–769 additional deaths during the 2003 UK heatwave (Donnelly et al. 2016; Stedman 2004). There have also been recent deaths during athletic events held in extreme temperatures, for example, the Belfast and London marathons (mostly competed by the general public) and also serious medical incidents concerning elite athletes, such as the infamous case of Jonathon Brownlee collapsing in Cozumel, Mexico in 2016 during the triathlon Grand Final and Scottish marathon runner Callum Hawkins during the 2018 Commonwealth Games held on the Gold Coast, Australia (BBC Sport 2016, 2018; BBC 2018; The Guardian 2018). As a result, and with predicted increases in global temperatures, the relevance of this research for both the health and safety of elite athletes and recreational exercisers is increased (Trundle et al. 2015). This study therefore addresses the need to examine how local meteorology and air quality can affect elite athletes to better inform event scheduling and safeguard both elite and recreational exercisers.

## Data and methodology

This research follows a similar approach to our previous investigation into the influence of local meteorology and air quality on the performance of the general public at parkrun events in Greater London (Hodgson et al. preprint). In this paper, the focus is on the 5-km running event, which requires maximal oxygen uptake (VO_2_ max, or the maximum amount of oxygen a person can utilise during exercise), to determine whether elite athletes are influenced by variations in local air quality and meteorological conditions. Furthermore, this allows for a direct comparison to our previous work examining the influence of meteorology and air quality on recreational runners over the same distance.

**Fig. 1:**
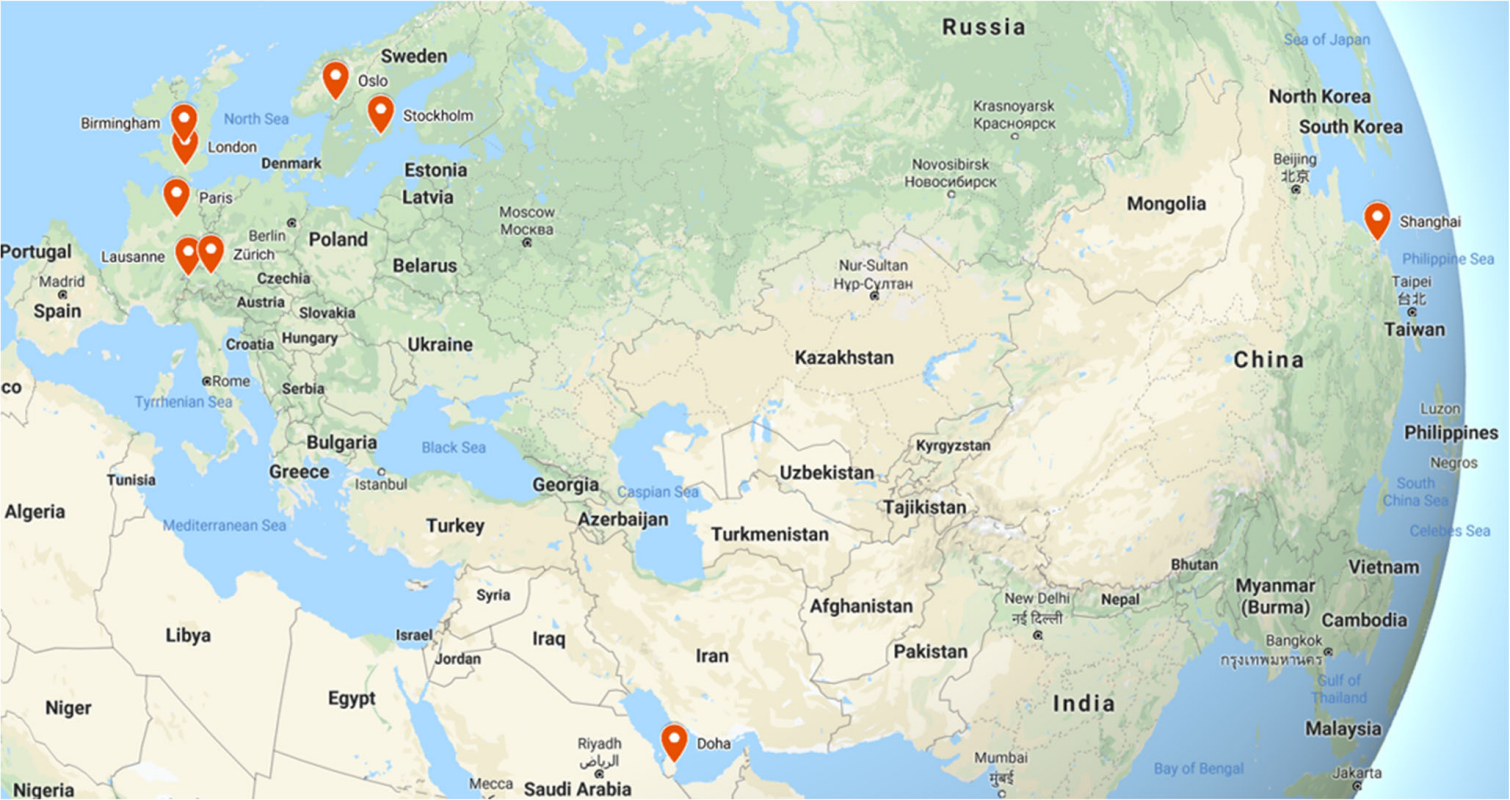
Map of Diamond League locations used in the analysis

Diamond League events are relatively consistent in their held locations over the season, travelling to various major cities, although there is a strong European presence. Finishing times for eight events/locations that have multiple years' worth of data have been collected from the IAAF Diamond League results archive, as well as a solitary event from Doha in 2010 (Fig. 1). Events in Rome, Eugene, Rabat, and Monaco, although hosting 5-km events, have not been included in this analysis due to a lack of readily available meteorological or air quality data. As well as finishing times of all 5-km participants, official start times of events and notation of whether it was a male or female event were also recorded to allow for accurate pairing of race times to local meteorological and air quality measurements, along with examination of male and female data subsets, as performed by Marr and Ely (2010). It is noted that Diamond League events do not necessarily have the same races for males and females on the same day, or even same year, for individual locations.

The meteorological data used for analysis has either been retrieved through the worldmet package in R (Carslaw 2018), or (where available) official data used by national monitoring bodies. Monitoring locations were selected due to their proximity to event locations and ability to provide hourly readings of temperature, relative humidity, and wind speed. Air quality and meteorological data was acquired from the following local authorities, MeteoSwiss (Switzerland), Oslo Kommune (Norway), AirParif (Frane), The Department for Environment, Food and Rural Affairs (UK) and The Swedish Meteorological and Hydrological Institute (Sweden) for data provision. Again, air quality monitoring sites are chosen for their proximity to Diamond League events to minimise the chance of spatial variation in pollutants influencing analysis and, where possible, includes hourly measurements of O_3_, NO_2_, PM_10_ and PM_2.5_. How representative the air quality data for the Diamond League event needs to be considered. O_3_ is generally a regional pollutant with less variability compared to NO_2_ and PM_2.5_, which are more likely to be influenced by local sources and may have greater potential for discrepancies between monitoring and event locations. For multiple linear regression analysis, PM_10_ has been removed from analyses due to its high correlation to PM_2.5_ and the higher association of the latter pollutant with deleterious health effects. A summary of the acquired data for Diamond League events, meteorology and air quality can be seen in Table 1.

**Table 1:** IAAF Diamond League events examined, along with the corresponding availability in local air quality and meteorology data. It is important to note that there is only a single (male or female) 5K race held at each event, e.g., London 2011 only has a female race, whilst 2013 is a male race

Event	Years	O_3_	NO_2_	PM_10_	PM_2.5_	Wind speed	Temp	RH
London	2011, 2012, 2014–2016, 2018	All years	All but 2012	All but 2015	All years	All years	All years	All years
Birmingham	2011, 2013, 2015, 2016	All years	All years	2015, 2016	2015, 2016	All years	All years	All years
Paris	2010–2015	All years	All years	No data	No data	2010–2014	2010–2014	2010–2014
Zurich	2010–2014, 2016–2018	2018	2018	2018	2018	All years	All years	All years
Oslo	2010–2016	All years	No data	All years	All but 2014	All years	All years	All years
Doha	2010	No data	No data	No data	No data	All years	All years	All years
Shanghai	2010–2018	No data	No data	No data	No data	All but 2015 and 2017	All years	All years
Stockholm	2010, 2011, 2014, 2016, 2018	All but 2018	All but 2018	No data	2010, 2011, 2016	All years	All years	All years
Lausanne	2011, 2013, 2015, 2017, 2018	2018	2018	2018	No data	All years	All years	All years

Each Diamond League event was paired with the closest meteorological and air quality monitoring station and the closest average hourly reading to the event time was used. All data was checked for normality, homogeneity and kurtosis prior to analysis and was logged where necessary. As per Helou et al*.* (2012) and Hodgson et al. (preprint), a correlation analysis between finishing times and control variables was performed for the whole data set, as well as individual events. This followed a preliminary analysis to investigate the role of gender due to the large differences between male and female finishing times. Next, linear regression was used to determine the extent to which the control variables influenced finishing times, as per previous marathon studies whilst multiple linear regression analysis examined the combined influence of meteorological and air quality variables on performance (Maffetone et al. 2017). Finally, posttest analyses were also performed using the following diagnostic tests: quantile–quantile, scale–location, fitted vs residuals, and Cook’s distance plots. To determine whether there were any significant differences between male and female events finishing times and the explanatory variables at the respective event times, a one-way ANOVA with suitable post hoc tests was performed. This was also used to determine whether there were differences between the nine events response and explanatory variables, and was again looked at as a complete dataset and male/female subsets. The mean finishing times of successfully completed races and explanatory variable figures were also determined to aid descriptive statistics and one way ANOVA comparisons and analysis.

## Results and discussion

### Overall performance analysis

**Fig. 2:**
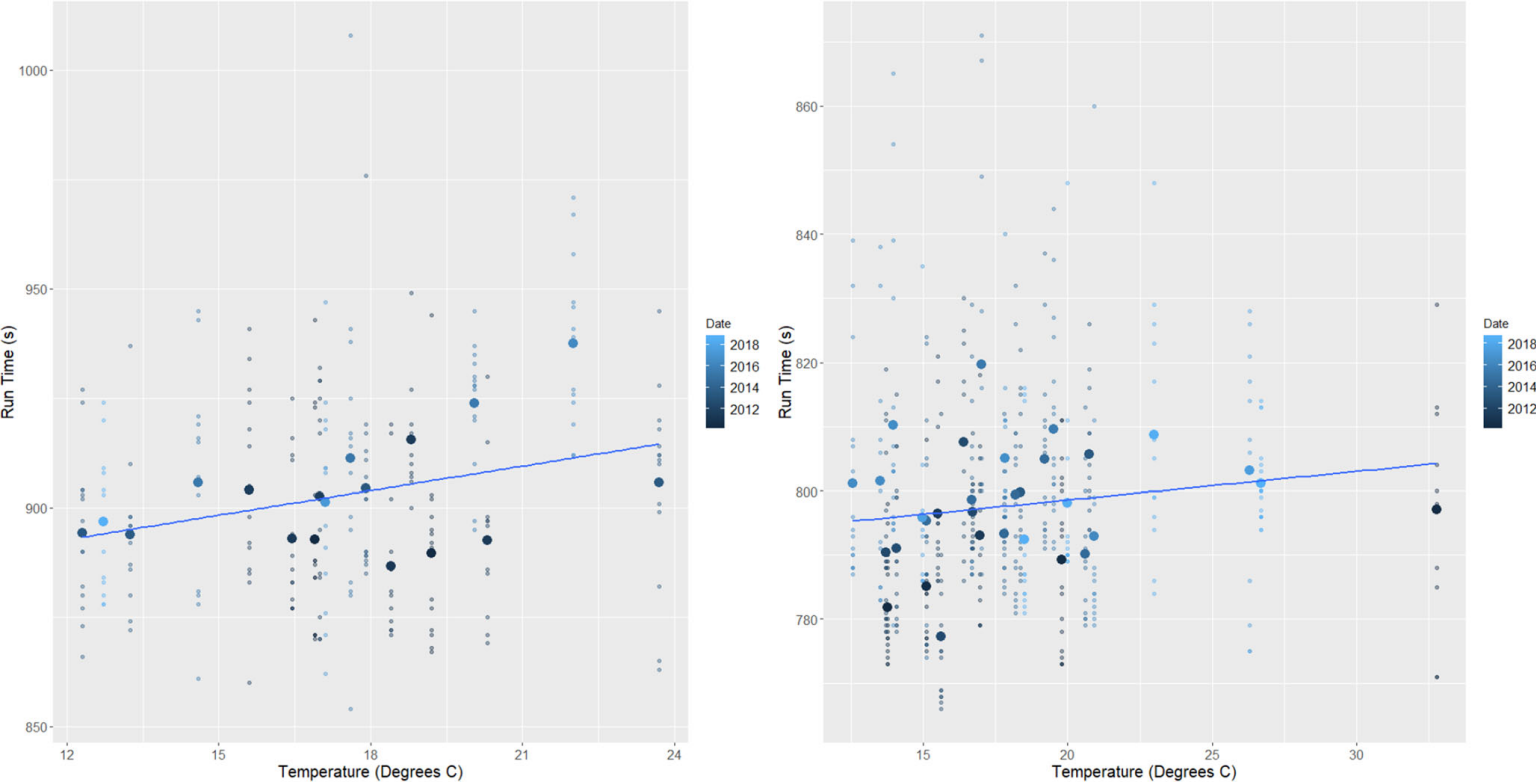
The effect of increasing temperatures on (left) female and (right) male races across the study period. The smaller points show the individual finishing times whilst the largest points are the mean time for each race

**Fig. 3:**
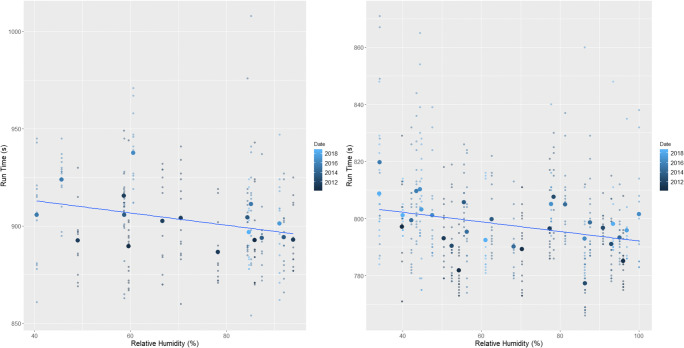
The effect of increasing relative humidity on (left) female and (right) male races across the study period. The smaller points show the individual finishing times whilst the largest points are the mean time for each race

**Fig. 4:**
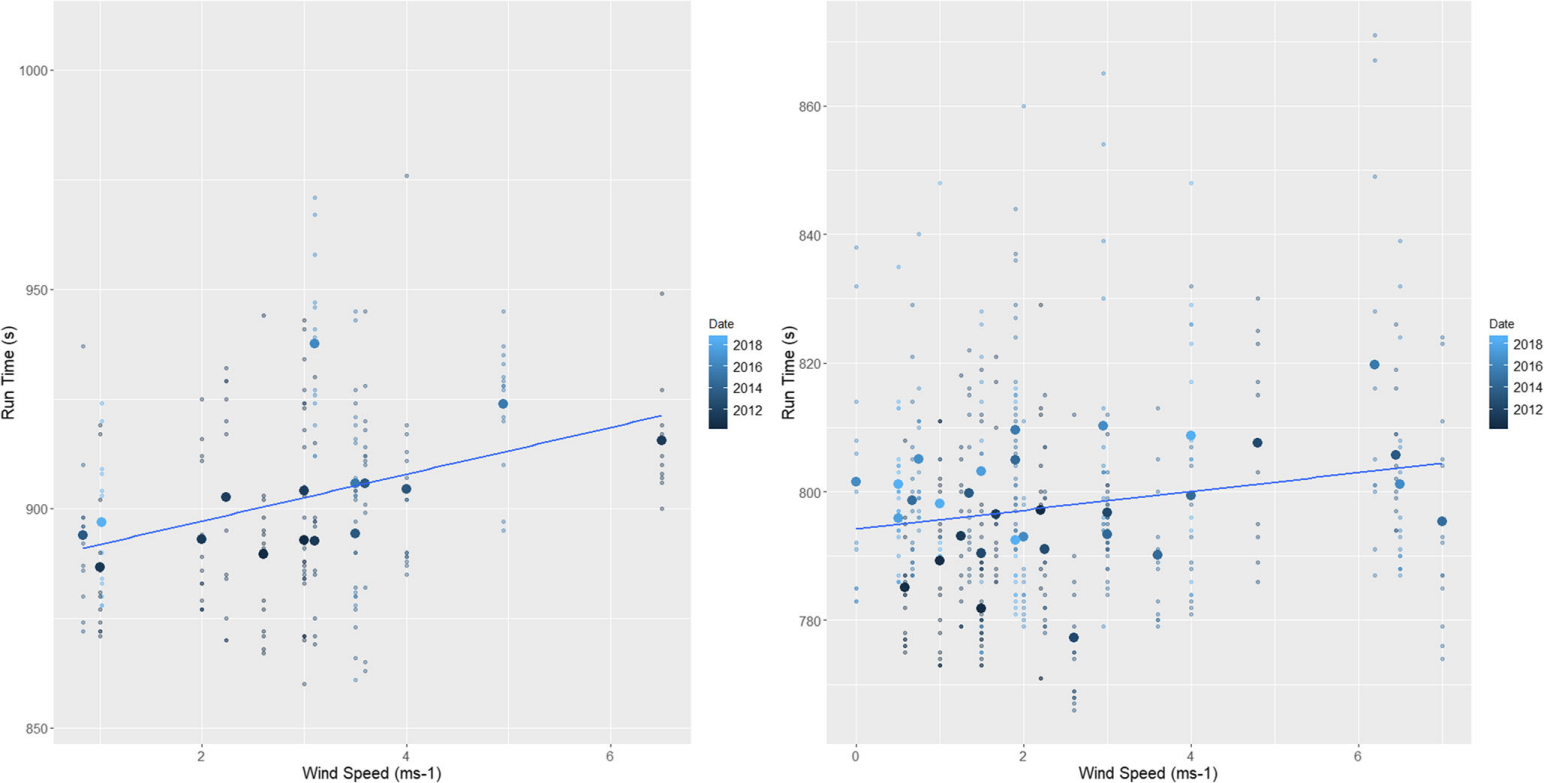
The effect of increasing wind speeds on (left) female and (right) male races across the study period. The smaller points show the individual finishing times whilst the largest points are the mean time for each race

Analysis showed that at both male and female events, higher wind speeds and temperatures resulted in slower finishing times whilst higher relative humidity saw correspondingly quicker events (*p*=<0.01) (Table 2 and Figs. 2, 3, and 4). The subsequent multiple regression analysis also indicated that higher temperatures slowed performances (*p*=<0.01 and *p*=0.06 for male and female events, respectively). As noted already, the influence of wind and temperature on performance is to be expected, whilst higher relative humidity is thought to reduce heat dissipation and consequently lead to slower finishing times (Casa 1999; Nadel 1990). Despite this, at both the male and female events, temperature and relative humidity are negatively correlated, which explains the relationship shown between relative humidity and finishing times. Temperature is therefore the more influential parameter on performance and athletes are able to run faster in cooler but more humid conditions (Bigazzi 2017). This has been previously shown by Daniels (2014), Helou et al. (2012) and Knechtle et al. (2019) examining marathon events, where each 5^o^C increase in temperature will decrease performance by up to 1.6%. This is likely due to changes in athlete’s circulatory and thermoregulatory systems to maintain a stable core body temperature (Casa 1999; Ely et al. 2007; Helou et al. 2012; Miller-Rushing et al. 2012; Nadel 1990; Nybo et al. 2014; Vihma 2010; Vugts 1997; Zhao et al. 2013). Athlete’s core body temperature is likely to increase at a quicker rate than the aforementioned marathon studies because of the higher intensity exercise being performed, and thus metabolic heat produced during shorter duration events (Cheuvront and Haymes 2001; Gasparetto and Nesseler 2020).

**Table 2:**
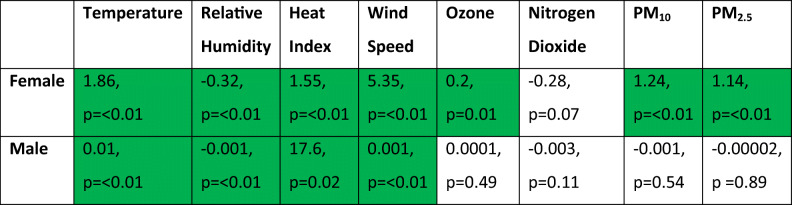
Linear regression coefficients and associated *p* values between female and male finishing times and associated explanatory variables

Green-shaded cells indicate statistical significance of < 0.05

The combined influence of temperature and relative humidity was also calculated as a heat index using the weathermetrics package in R Studio (Anderson et al. 2013). For the male analysis, correlation, regression and multiple linear regression (heat index + wind speed) was detrimental to performance (*p*=<0.02). For female athletes, heat index also contributed to slower running times for correlation and regression (*p*=<0.01) and when combined with increased wind speeds was also detrimental (*p*=0.06). Consequently, the combined influence of variables is likely to increase core body temperatures of athlete’s and limit heat dissipation, with heat stress being cited as a concern for health during exercise, particularly under future climate change scenarios (Miller-Rushing et al. 2012; Morici et al. 2020). These results also support those of Vihma (2010) and Knechtle et al. (2019) who suggested that despite difficulties in quantifying the effect of wind on performance due to its variable nature, head- and cross-winds will reduce running speeds. This is likely to be found at Diamond League events held on a standard 400 m athletics track with potentially less of the distance covered per lap being assisted with a tailwind. Furthermore, any potential tailwind is unlikely to benefit athletes in this circumstance overall as research has shown that head- and cross-winds are more detrimental than tailwinds are beneficial (Davies 1980). The slightly reduced effect of heat index and wind speed on female athletes compared to male may be due to gender differences in heat dissipation. Core temperature control is greater for females due to a generally larger surface area to mass ratio and higher subcutaneous fat content (Gagnon et al. 2009; Kaciuba-Uscilko and Ryszard 2001). Female body mass is also likely to be lower due to physiological differences in stature, musculature, and body fat percentages: which has been shown to be advantageous for running under increased temperatures (Cheuvront et al. 2002; Cheuvront et al. 2005; Marino et al. 2000; Zouhal et al. 2011). Females also have a higher running economy than males, which would lead to reduced heat production and less performance decreases over time (Billat et al. 2001). Consequently, it can be hypothesised that under elevated heat index conditions, female athletes are producing less metabolic heat and also being cooled sufficiently by the wind to maintain a stable core temperature and suffer less of a performance decrease compared to their male counterparts (Maughan et al. 2007a; Maughan et al. 2007b; Maughan 2010).

**Fig. 5:**
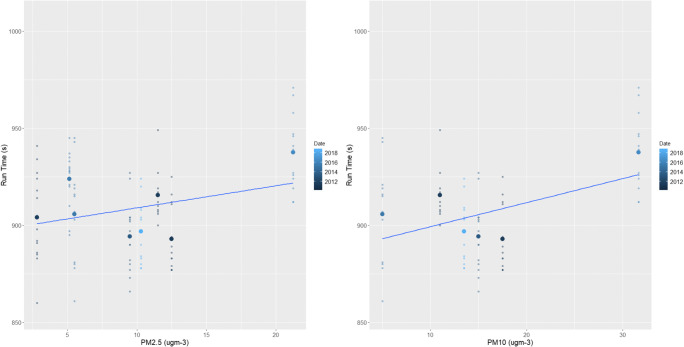
The effect of increasing PM_2.5_ (left) and PM_10_ (right) male races across the study period. The smaller points show the individual finishing times whilst the largest points are the mean time for each race

**Fig. 6:**
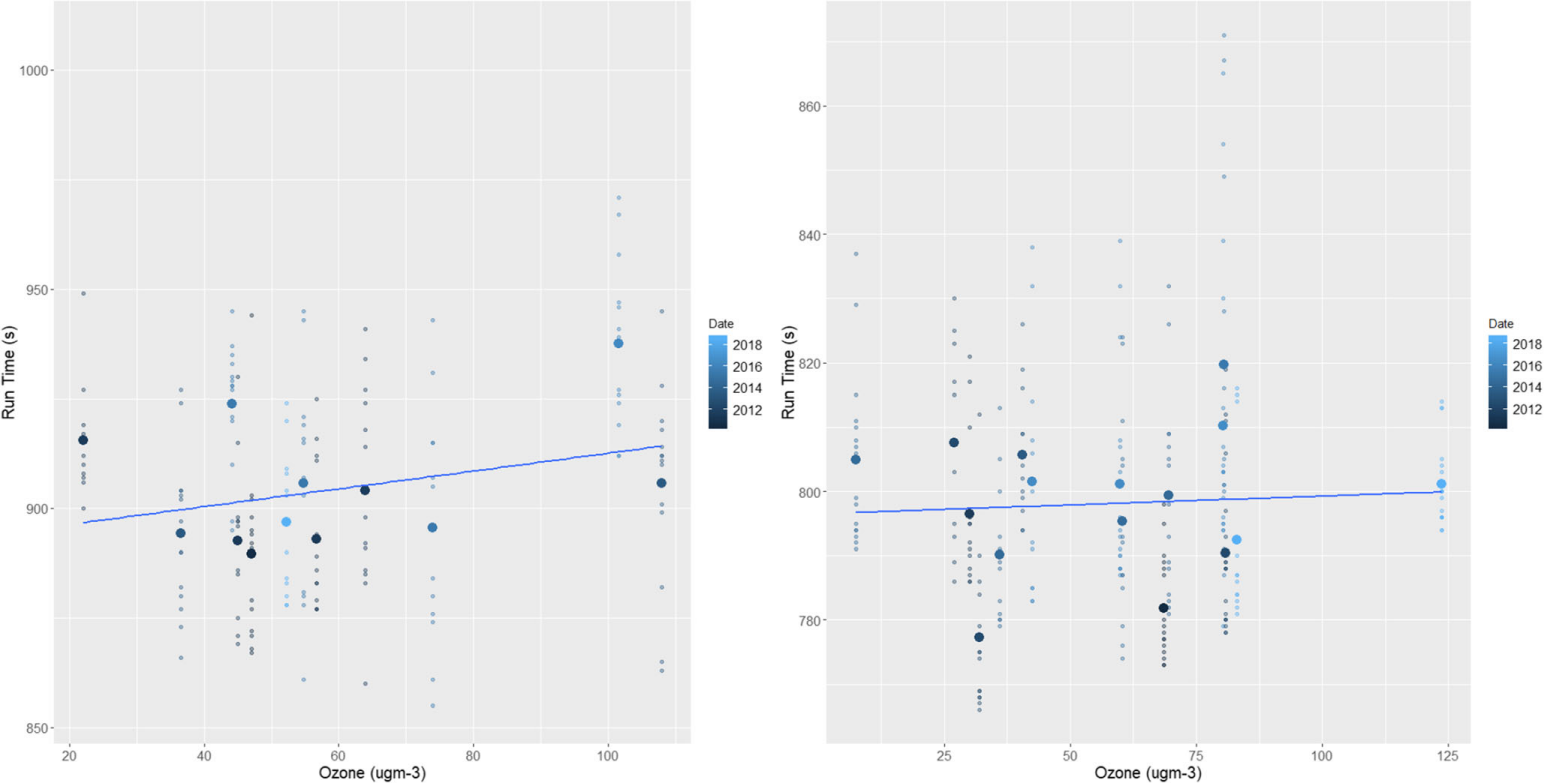
The effect of increasing ozone on female (left) and male (right) races across the study period. The smaller points show the individual finishing times whilst the largest points are the mean time for each race

In addition to significant relationships with temperature, relative humidity and wind, female races also returned several other significant results (Figs. 5 and 6). In terms of air quality, O_3_ and both PM_2.5_ and PM_10_ caused female athletes to also produce slower finishing times (*p*=<0.01, Table 2). The known impacts of air quality on physical health-decreasing lung and cardiovascular function, irritation of the respiratory system, chest tightness and reduced arterial pressure and vasodilation-are likely to have contributed to these results in female performances (Carlisle and Sharp 2001; McKenzie and Boulet 2008; Rundell and Caviston 2008; Rundell 2012). This is through a reduction in oxygen uptake and VO_2_ max and increased perceived exertion levels (Florida-James et al. 2011; Giles et al. 2014; Giles et al. 2018; Kargarfard et al. 2015). It should also be noted that the above air quality impacts would be heightened for those with cardiorespiratory conditions such as asthma (Cutrufello et al. 2011; Rundell et al. 2008). As asthma and exercise induced asthma is widespread within the elite athlete demographic, this may be a contributor to the results presented and should be carefully considered for elite sports events in the future (Folinsbee et al. 1994; Helenius et al. 1997; Langdeau et al. 2000; Langdeau and Boulet 2001; Lippi et al. 2008; McCreanor et al. 2007; McKenzie and Boulet 2008; Rundell 2012; Turcotte et al. 2003; Weinmann et al. 1995).

Overall, these findings add additional insight into the potential role of air quality in previous research that has shown temperature to be the biggest environmental influencer on athletic performance (Ely et al. 2007; Helou et al. 2012; Marr and Ely 2010). Similar results for the male races were not observed. The reason as to why female athletes are more influenced by air quality than male athletes is currently unclear but was also shown by Marr and Ely (2010) with PM_10_ increases of 10μg/m^3^ reducing performance by 1.4%.

### Individual events

Events at the nine individual locations were also analysed to determine whether individual locations showed relationships between finishing times and the explanatory variables. A one-way ANOVA was subsequently performed to determine whether differences in finishing times across the nine events could be explained by variations in the explanatory variables.

The mean finishing times of the male and female events are shown in Table 3. The fastest two events for both genders were Paris and Oslo, respectively. Zurich is the third quickest female event and fourth for the male subset, with Shanghai being fourth and third quickest. With the exception of Doha’s solitary race, the other four locations are slower than the mean finishing times. Interestingly, London and Birmingham are the second slowest and slowest events of the Diamond League series, the latter being over 35 s slower than the mean female time and nine seconds for male events. For the female events, the ANOVA further shows that London is significantly slower than the four quickest events (*p*=<0.01) and Birmingham is slower than the top four events, as well as Stockholm that is also slower than the mean finishing time (*p*=<0.04). ANOVA analysis of the men’s events also shows a number of significant differences. Again, Birmingham and London are significantly slower than the quickest two events (*p*=<0.01) and Paris is also significantly quicker than Stockholm and Lausanne (*p*=<0.01).

**Table 3:** Mean finishing times of the nine Diamond League events

Event	Female mean finishing time (s)	Male mean finishing time (s)
Paris	895.92	783.23
Oslo	897.36	792.97
Zurich	897.84	795.41
Shanghai	899.88	795.18
Doha	N/A	797.09
All events	902.55	797.7
Stockholm	904.14	797.89
Lausanne	N/A	800.09
London	920.42	805.69
Birmingham	937.62	806.74

Table 4 below shows the linear regression results for the gender analysis of all the Diamond League events, followed by additional discussion of each location.

**Table 4:**
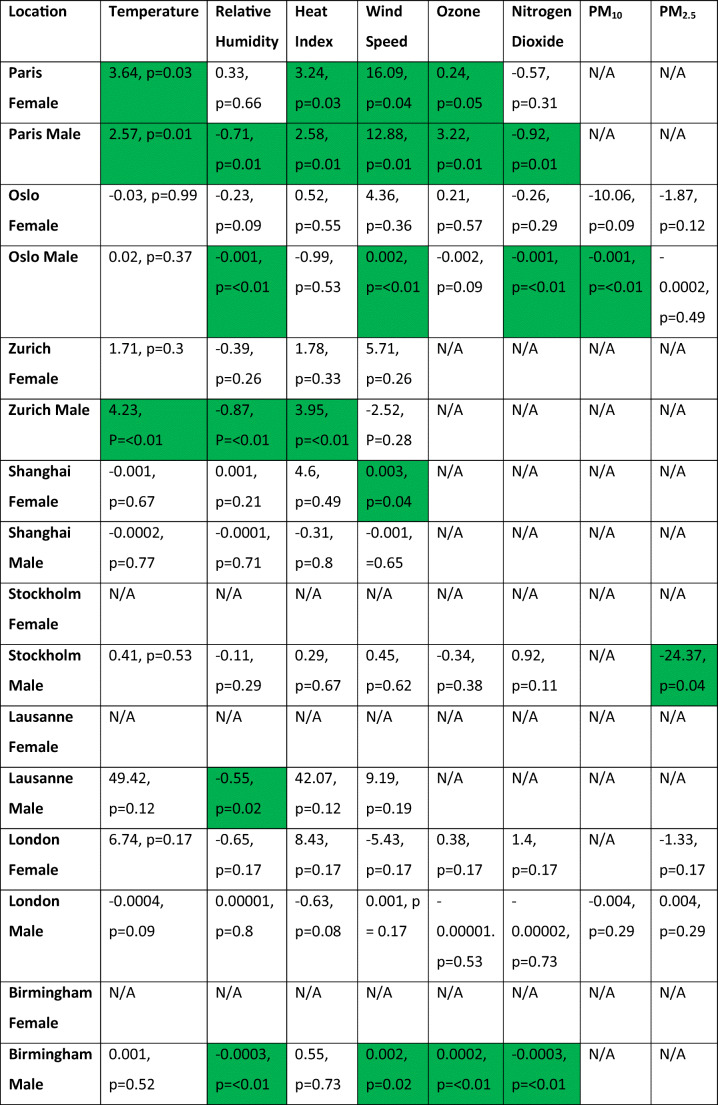
Linear regression coefficients and *p* values between finishing times and explanatory variables

Green-shaded cells indicate statistical significance of <0.05

#### Birmingham

**Fig. 7:**
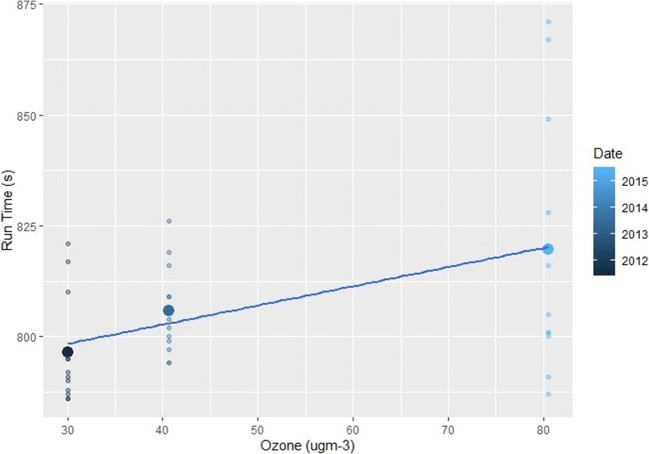
The effect of increasing Ozone levels on male races held in Birmingham. The smaller points show the individual finishing times whilst the largest points are the mean time for each race

Birmingham is the slowest event of the Diamond League calendar and the male subset showed a number of significant results. More O_3_ and windier conditions produced slower results (Fig. 7), whilst higher levels of NO_2_ and relative humidity were often conducive to quicker results (*p*=<0.02). This suggests that, in the case of Birmingham, the irritant qualities of O_3_ can play a detrimental role during the 5 km event. In contrast, but similar to results of Hodgson et al. (preprint), NO_2_ slightly improved finishing times, potentially linked to the reaction between O_3_, VOCs, NO_2_ and sunlight, with higher NO_2_ levels occurring under lower temperatures.

Birmingham showed significant and detrimental relationships with O_3_, PM_10_ and PM_2.5_ and temperature slowing finishing times (*p*=<0.01). Conversely, higher levels of NO_2_ saw improved finishing times, likely due to the lower temperatures found under those conditions and highlighted previously (*p*=<0.01). Additionally, the highest mean levels of O_3_ are found at these events, along with the second lowest NO_2_ levels. Based upon previous research, these high O_3_ and low NO_2_ levels could be contributing to slower finishing times (Ely et al. 2007; Helou et al. 2012; Marr and Ely 2010). Furthermore, the city registers the second highest PM levels on race times and is also the windiest event, significantly more so than several calmer locations (*p*=<0.02). Based upon these ANOVA and regression results, it appears that high O_3_ and PM levels, coupled with low NO_2_ and high wind speeds, is contributing to the slower finishing times at the Birmingham races.

#### Doha

Doha only had one event and was therefore not analysed independently.

#### Lausanne

No air quality data was available for the study period in Lausanne which also only had male events to analyse. There were no significant relationships between finishing times and temperature, relative humidity, wind speed or heat index. Generally, Lausanne has low wind speeds and relative humidity, but is the warmest event once Doha is discounted. Consequently, it is suggested that the high temperatures and low cooling wind speeds are potentially contributing to Lausanne’s mean finishing time being the third slowest—although this cannot currently be statistically proven and only provides a guideline to the impact of local meteorology on athletic performance.

#### London

For the gender subsets of events held in London, there were no significant relationships shown. Despite this, London, along with Birmingham, was the slowest event for male and female athletes, significantly so compared to the other six locations (*p*=<0.08). The conditions athletes were competing in also included the highest O_3_, NO_2_, PM_10_ and PM_2.5_ levels. For PM, London’s levels were significantly the highest (*p*=<0.01). These combined pollutants and their irritant qualities are likely to have slowed performances to an extent in the London events (Cutrufello et al. 2011; EEA 2013; Rundell and Caviston 2008; Rundell et al. 2008; Rundell 2012).

For female races, London is the second slowest event, but has significantly low O_3_ levels (*p*=<0.02) and higher NO_2_ (*p*=<0.01). Along with Stockholm, London has the lowest levels of PM (*p*=<0.09) and is the windiest event. From this, there is not necessarily a clear picture as to why London’s female races are on the slower end of the spectrum, due to the low O_3_ and PM levels. The main explanation could be the high wind speeds of 5.6 ms^−1^. Although this is not significantly higher than other locations, it may be enough to contribute to the decreased running speeds as shown by Davies (1980).

#### Oslo

**Fig. 8:**
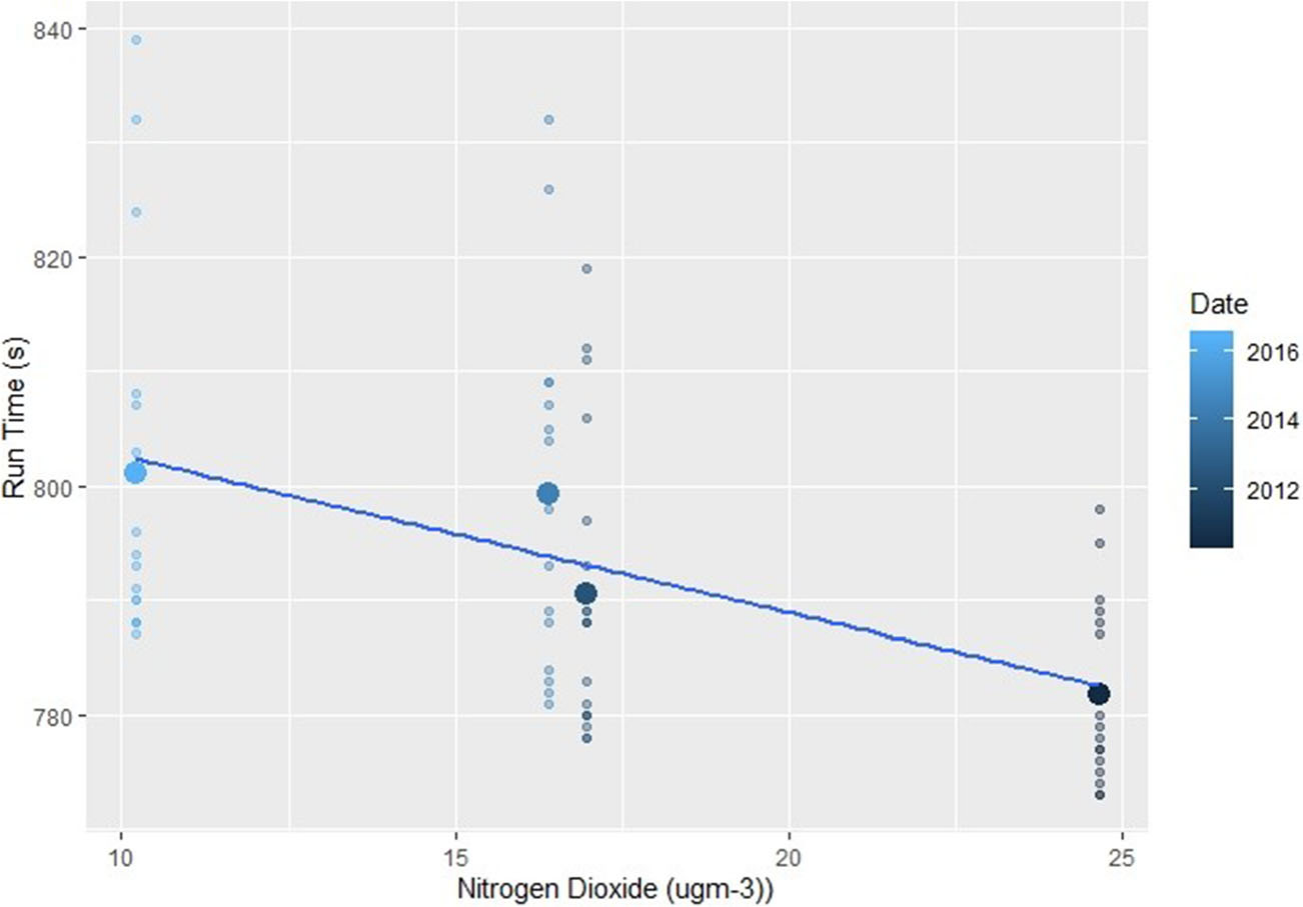
The effect of increasing NO_2_ levels on male races held in Oslo. The smaller points show the individual finishing times whilst the largest points are the mean time for each race

The male subset in Oslo only showed three significant results, generally, finishers are quicker under elevated NO_2_ (Fig. 8), relative humidity and PM_10_ concentrations (*p*=<0.01). For female events, higher relative humidity and PM_10_ produced quicker results (*p*=0.09). Oslo is the second quickest event for male and female races, and often has cooler and more humid conditions, which would promote fast times and correlates well with previous research (Hodgson et al. preprint). The PM results are not to be expected due to the irritant nature of the pollutant, however (EEA 2013).

#### Paris

The male subset at Paris also showed a number of significant results. Higher O_3_, temperatures and windy conditions produced slower results, whilst increased levels of NO_2_ and relative humidity improved performances (*p*=<0.02). Heat index results also suggest that under combined high temperatures and relative humidity performances are slower (*p*=0.01). The female subset also showed the same impacts of those three variables (*p*=<0.05) and mirrors previous research findings (Casa 1999; Ely et al. 2007; Helou et al. 2012; Hodgson et al. preprint).

From ANOVA analysis, Paris is the quickest event for male and female mean times when compared to a number of the slower locations. Paris has the lowest O_3_ and highest NO_2_ levels, significantly so when compared to the highest and lowest locations, respectively (*p*=<0.01). Consequently, this may explain why the male event at Paris is the quickest of the nine. However, for female events, pollutant and meteorological conditions do not show any significantly high or low values for Paris.

#### Shanghai

**Fig. 9:**
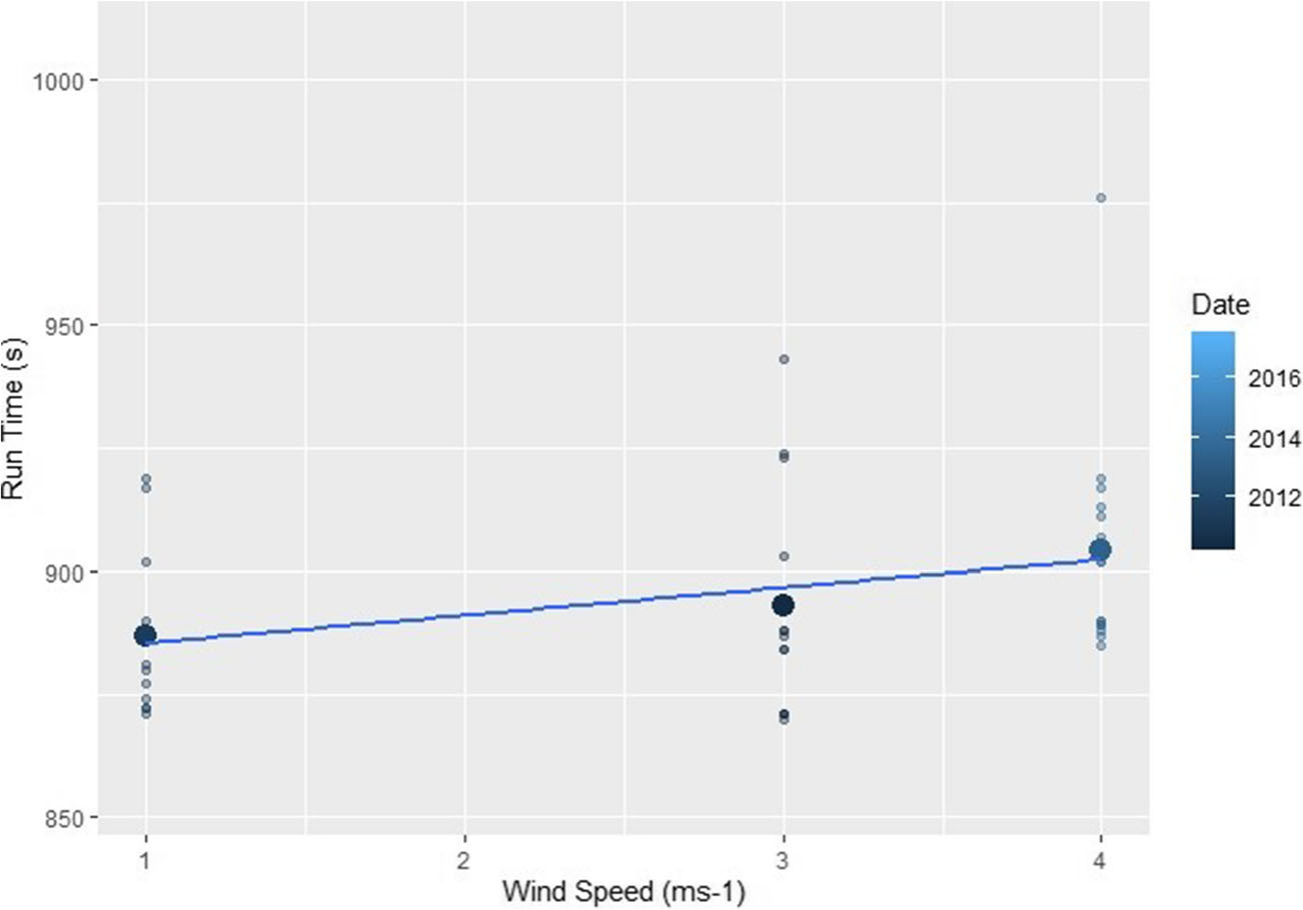
The effect of increasing wind speeds on female races held in Shanghai. The smaller points show the individual finishing times whilst the largest points are the mean time for each race

Shanghai only has meteorological data available, and the male subset showed no significant relationships whilst female events are slower under windier conditions (*p*=0.04, Fig. 9). This reduction in running speed under higher wind speeds is to be expected (Davies 1980). The lack of significant relationships with temperature or relative humidity could be attributed to Shanghai not having particularly high or low temperatures, instead having the highest relative humidity of around 90%. Shanghai is also around the mean run time of all events, suggesting that the meteorological conditions are not extreme enough, in a detrimental capacity, to have a clear impact on the overall finishing times compared to other locations.

#### Stockholm

For the male subset at Stockholm, only PM_2.5_ had a significant relationship with finishing times, improving them under elevated levels (*p*=0.04). There were no female events to analyse. Despite this unusual relationship with PM_2.5_, Stockholm generally has the lowest readings at its events (*p*=<0.03), as well as significantly low NO_2_ levels (*p*=<0.05). O_3_ concentrations are also low. Temperature, relative humidity, and wind speeds are not particularly extreme in either direction, suggesting that other factors may be influencing performance or that in this instance, the meteorological and air quality conditions are not great enough to impact elite athletic performance.

#### Zurich

**Fig. 10:**
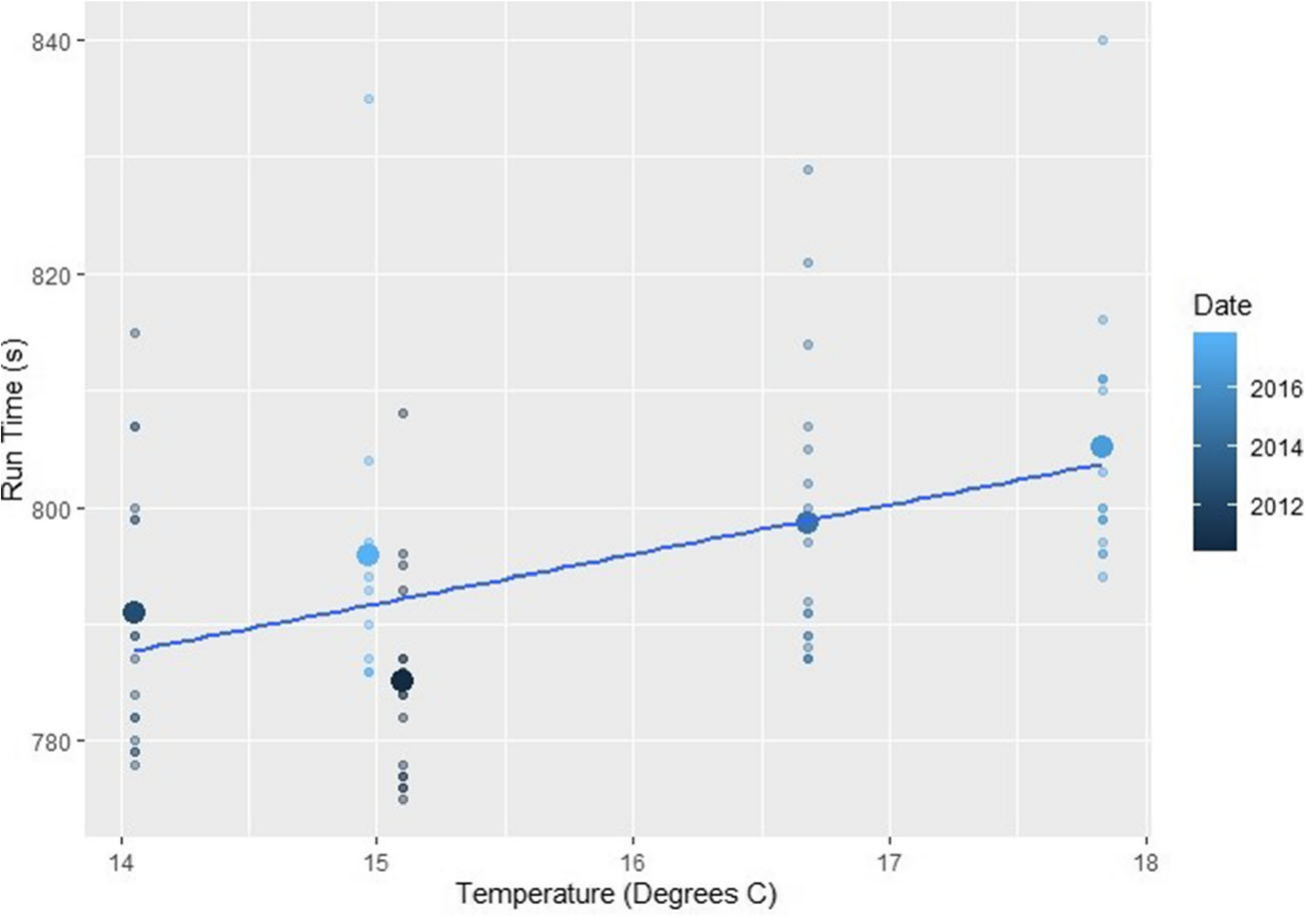
The effect of increasing temperatures on male races held in Zurich. The smaller points show the individual finishing times whilst the largest points are the mean time for each race

Zurich’s male subset conformed to previous research with higher temperatures slowing performances, whilst relative humidity was beneficial (*p*=<0.01, Fig. 10). Heat index was also detrimental to performance (*p*=<0.01). The female subset showed no significant results. Generally, Zurich is one of the colder and more humid events, as well as having the lowest wind speeds (*p*=<0.01). This could suggest that under ‘normal’ conditions Zurich is below an optimum race temperature for sufficient muscular performance as well air density being higher and thus providing increased resistance. Also, without air quality data, full conclusions as to the influence of variables on performance cannot be drawn.

## Summary and practical applications

Overall, there is a lot a variability in results when examining different locations that host Diamond League events. This is to be expected with distances between events and monitoring locations and the highly spatial variability of pollutants, particularly PM and NO_2_ (Duyzer et al. 2015; Ferradas et al. 2010; Kaur et al. 2005, 2007). With respect to meteorology, windier conditions and higher temperatures are most detrimental to performance, with the latter inversely correlated with relative humidity and quicker finishing times coming from events with low temperatures and high relative humidity. With regard to track surfaces, Diamond League venues have to meet and maintain IAAF standards, so although there might be some small difference between track age and composition there is unlikely to contribute to vastly different running performances.

Paris and Birmingham show the most promising results. Paris has the quickest finishing times, whilst also having the lowest O_3_ conditions and second highest NO_2_ levels. Additionally, events are held under lower than average temperatures and regression results suggest that low O_3_, temperatures and higher NO_2_ levels will result in the quickest times. For Birmingham, the slowest event, high O_3_, PMs and temperatures result in slower performances, and events are generally held under high levels of the first two variables. This potentially suggests that despite elite athletes training for high temperatures, they are not as well conditioned to high pollution levels, particularly PM, which will generally be low at high altitude training locations.

Despite attention being drawn to air quality concerns at previous Olympic Games held in Athens, Beijing and Rio and various methods being used by host nations to improve conditions prior to events, there has been no consideration of the impact of environmental conditions on performances (De La Cruz et al. 2019; Donnelly et al. 2016; Florida-James et al. 2011; Wang et al. 2009). It is hoped that this research will prompt further investigation into the influence of air quality and meteorology variables on elite athletic performance and health, as well as scheduling events at times when pollution is lowest not only for athletes, but also spectators who will also be influenced by air pollution. This has previously occurred with regard to temperature with the now 2021 Tokyo Olympics endurance events being scheduled to avoid the warmest of temperatures, but additional mitigation measures could be implemented (BBC Sport 2019a, 2019b). For instance, road closures along and around events, as well as the provision of public, active, or ‘green’ transport options for spectators and officials, may help reduce pollution levels at the event whilst Kosaka et al. (2018) suggested that increased shading along the Tokyo marathon route may reduce the likelihood of heat stress related medical incidents (Morici et al. 2020). This is especially so when research has highlighted the positive relationship between increased temperatures, associated medical incidents and the total number of ‘did not finish’ participations (Carlstrom et al. 2019; Khorram-Manesh et al. 2020; Schwabe et al. 2014).

Furthermore, with over half the world’s population currently living in urban areas, the majority of whom are under air quality conditions that exceed the World Health Organisation’s guidelines, consideration of not only elite athletic event locations and timings but also recreational exercisers should be considered (Hewitt et al. 2020; Marmett et al. 2020). Prior to the COVID-19 pandemic, participation at parkrun events (weekly, timed 5000 m running events) and mass participation runs across a variety of distances were incredibly popular (Scott 2021; Yankelson et al. 2014): parkrun reached over six million registered runners in 2019, Helou et al*.* (2012) showed that marathon entries increased over a ten year period and Brocherie et al*.* (2015) also found a 26% increase in the American running population between 2007 and 2012 (Parveen 2019). This has the potential for a greater number of people being exposed to harmful pollution levels, putting greater strain on population health, associated health services and productivity (Kumbhakar et al. 2021). This highlights the need for additional research into the effect of air pollution and meteorology on athletic performance and health, as well as the best methods to mitigate detrimental outcomes at local and international sporting events and during recreational exercise.

## Conclusions

Following on from previous research into marathon studies and examination of parkrun events, this research has looked to examine the influence of meteorology and air quality on elite 5-km running performance at Diamond League events. Although analysis results vary across event locations, the influence of meteorological parameters correlates well with previous research and suggests that they are the most influential on elite athletic performance. Of pollutants, O_3_ appears to be the greatest influencer on performance, with NO_2_ seeing improved finishing times. This correlates with previous research and is likely linked to the relationship between these two pollutants, sunlight (temperature) and VOCs. Although specifically aimed at elite athletes, this study helps support research in the air quality and physical activity field, as well as providing insights into the timing of events for both elite and recreational athletes to best minimise potentially detrimental impacts on athletic performance as well health related effects of air pollution.

## Data Availability

Datasets generated and used during the study are available in the DANS repository: 10.17026/dans-xmr-tnx5.
